# “What about building 7?” A social psychological study of online discussion of 9/11 conspiracy theories

**DOI:** 10.3389/fpsyg.2013.00409

**Published:** 2013-07-08

**Authors:** Michael J. Wood, Karen M. Douglas

**Affiliations:** School of Psychology, Faculty of Social Sciences, University of KentCanterbury, UK

**Keywords:** persuasion, online discussion, social influence, archival research, conspiracy theories

## Abstract

Recent research into the psychology of conspiracy belief has highlighted the importance of belief systems in the acceptance or rejection of conspiracy theories. We examined a large sample of conspiracist (pro-conspiracy-theory) and conventionalist (anti-conspiracy-theory) comments on news websites in order to investigate the relative importance of promoting alternative explanations vs. rejecting conventional explanations for events. In accordance with our hypotheses, we found that conspiracist commenters were more likely to argue against the opposing interpretation and less likely to argue in favor of their own interpretation, while the opposite was true of conventionalist commenters. However, conspiracist comments were more likely to explicitly put forward an account than conventionalist comments were. In addition, conspiracists were more likely to express mistrust and made more positive and fewer negative references to other conspiracy theories. The data also indicate that conspiracists were largely unwilling to apply the “conspiracy theory” label to their own beliefs and objected when others did so, lending support to the long-held suggestion that conspiracy belief carries a social stigma. Finally, conventionalist arguments tended to have a more hostile tone. These tendencies in persuasive communication can be understood as a reflection of an underlying conspiracist worldview in which the details of individual conspiracy theories are less important than a generalized rejection of official explanations.

“The Internet was made for conspiracy theory: it is a conspiracy theory: one thing leads to another, always another link leading you deeper into no thing and no place.”

(Stewart, 1999, p. 18).

Conspiracy theories, defined as allegations that powerful people or organizations are plotting together in secret to achieve sinister ends through deception of the public (Abalakina-Paap et al., 1999; Wood et al., 2012), have long been an important element of popular discourse. With the advent of the Internet, they have become more visible than ever. Although the psychological literature on conspiracy belief has a relatively short history, with most of the relevant research having been conducted only within the past twenty years, it has revealed a great deal regarding individual differences between those who generally believe conspiracy theories (whom we call “conspiracists”) and those who prefer conventional explanations (whom we call “conventionalists”). Conspiracy beliefs have been shown to be positively correlated with mistrust of other people (Goertzel, 1994) and authorities (Swami et al., 2010); feelings of powerlessness and low self-esteem (Abalakina-Paap et al., 1999); superstition, beliefs in the paranormal, and schizotypy (Darwin et al., 2011); a perceived lack of control (Hamsher et al., 1968; Whitson and Galinsky, 2008); a Machiavellian approach to social interaction (Douglas and Sutton, 2011); and openness to experience (Swami et al., 2010; but see Swami et al., 2011).

At the present time, questionnaire-based investigations of individual differences make up the bulk of the existing research, although experimental approaches are emerging (e.g., Douglas and Sutton, 2008; Jolley and Douglas, 2013). A fairly recent development in the field has been an acknowledgement that in addition to trait-like variables and transient psychological states, ideologies and broad belief systems play a substantial role in conspiracy theory belief. For example, in an examination of conspiracy theories regarding an alleged cover-up of the divinity of Mary Magdalene and the bloodline of Christ, Newheiser et al. (2011) demonstrated that the plausibility of these theories hinged largely on broader beliefs about the world. People with traditional Christian beliefs were likely to reject such theories out of hand, while those with a more New Age approach were much more receptive. In a similar vein, Lewandowsky et al. (2013b) demonstrated that rejection of climate science (though not explicitly conspiracist) is determined in part by ideological concerns, with libertarian free-market ideology, apparently predisposing people to believe that anthropogenic global warming is an unscientific hoax. It is clear, then, that individual conspiracy theories or related counter-normative explanations can seem more or less likely depending on how they comport with other beliefs held by the audience.

Some researchers have gone further, proposing the existence of a conspiracist worldview—a belief system conducive to conspiracy beliefs in general (e.g., Goertzel, 1994; Swami et al., 2010; Wood et al., 2012). This proposal stems primarily from the finding that beliefs in unrelated conspiracy theories tend to intercorrelate: for example, someone who believes that Princess Diana was deliberately assassinated is also more likely to believe that the moon landing was a hoax. Indeed, Wood et al. (2012) demonstrated that even beliefs in directly contradictory conspiracy theories were positively correlated with one another, indicating that conspiracy beliefs may be held together not by direct agreement with one another, but by mutual agreement with higher-order beliefs about the world. One particularly important element of the conspiracist worldview is thought to be a generalized opposition to official or received narratives. In this view, conspiracy belief is not about believing in particular alternative theories, but in disbelieving in whatever the official story is. This tendency has been informally noted by Dean (2002), who described most conspiracy theories as “bits and pieces without a plot… [that] fail to delineate any conspiracy at all. They simply counter conventional narratives with suspicions and allegations that, more often than not, resist coherent emplotment” (p. 92). Likewise, Clarke (2007) observed that conspiracy theories are often extremely vague, particularly in the Internet age.

If this is the case, then for people who hold a conspiracist worldview, the specifics of a conspiracy theory are less important than its identity as a conspiracy and its opposition to the official explanation. The important element is that those in power are lying and cannot be trusted, and that they are covering up something sinister. Opposition to officialdom, in this sense, parallels the generalized prejudice that Adorno et al. (1950) found to be strong enough to overcome contradictions between different anti-Jewish stereotypes. More than being a specific belief that Jews are overly secretive or overly intrusive, anti-Semitism appears to be more of a general belief that Jews are generally unpleasant people. Likewise, conspiracy theory belief appears to be more of a negative belief than a positive one—it is more concerned with saying what the cause of a condition or event was *not* (i.e., whatever the official explanation is) than with putting forward a specific alternative account.

An opportunity to test this idea presents itself in the form of observation of online discourse. In spite of, or perhaps because of, the lack of mainstream public acceptance for their theories, many conspiracists, both prominent and otherwise, appear to see themselves as having a duty to spread their views to the public at large. They often exhort the unthinking masses to “wake up” (e.g., Crane, 2008; Byers, 2009; Icke, 2012). This is a reasonable reaction: given a belief that people's lives are being manipulated by malevolent forces beyond their control, most would probably agree that trying to spread the word about that fact is a good idea. Outspoken conventionalists, such as those in the “skeptic” movement (e.g., Randi, 1982; Sagan, 1995; Shermer, 1997; Novella, 2009), find most conspiracy theories to be misguided at best and destructive at worst, and so make a point of arguing against them in the public sphere.

This discussion is voluminous and highly visible in many arenas, perhaps none more so than news website comment sections. Articles about topics for which popular conspiracy theories exist, such as 9/11, the moon landing, and vaccines, can have tens of thousands of comments, most of which are devoted to advancing or refuting allegations of conspiracy. These comments are often archived along with the associated articles for months or years afterward, which provides an excellent opportunity for archival research to give some insight into the thoughts and beliefs of those writing them (e.g., Fat et al., 2012; Loke, 2012; Sisask et al., 2012).

The present study consists of an examination of a large number of conspiracy theory-related persuasive comments on news stories. Such analysis of online discourse as a method of examining psychological states has increased in prominence as the Internet has become a more popular place to discuss one's ideas. The subject and pace of online discussion has been shown to be a more or less reliable barometer of public concern over social issues (Roberts et al., 2002; Scharkow and Vogelgesang, 2011), and emotional reactions expressed online can be used to consistently predict political approval ratings (Gonzalez-Bailon et al., 2012). Quantitative analysis of online discussion has also been used to gain insight into the social psychology of groups with fringe views (Douglas et al., 2005), attitudes toward Tourette's Syndrome (Fat et al., 2012), and racial views (Loke, 2012). Qualitative research on online discourse has been more common, including a study demonstrating the evolution of conspiracy theories over time in response to evidence (Lewandowsky et al., 2013a). In the context of conspiracy theories in particular, there are several advantages to content analysis of online commentary. The self-selective nature of online communication allows for the collection of a great deal of data regarding opinions that may be held by only a minority of people; moreover, the degree of anonymity facilitates the honest expression of opinions that might not be held in high social esteem elsewhere (e.g., Douglas et al., 2005; Loke, 2012).

There are some caveats associated with analyzing persuasive comments in particular. While external validity may benefit from observing behavior in a naturalistic setting, there is some degree of uncertainty regarding the internal validity of any conclusions drawn from such methods. Most obviously, there is the issue of to what degree the content of persuasive communications reflects the properties of the author rather than the demands of the situation. Rather than faithful representations of internal psychological processes, commenters' methods of argumentation might instead reflect strategic considerations regarding the audience, the venue, and the subject matter. While self-presentation is very often a concern in psychological research, even in laboratory settings, such demands may be especially salient in a situation where one's goal is implicitly (or even explicitly) to persuade others rather than to provide an honest and straightforward account of one's beliefs. Indeed, some research has shown that people do adapt their persuasive techniques according to their knowledge of the audience and the subject (Friestad and Wright, 1999; Douglas et al., 2010; Vogel et al., 2010).

The question of whether we can expect persuasive communication to accurately reflect inner psychological processes is not easily answerable, as the effect of lay persuasive knowledge on generation of persuasive arguments is fairly sparse. While there is a substantial body of research on lay persuasive knowledge, the vast majority of it focuses instead on how such knowledge affects susceptibility to the persuasive messages of others. However, it is well-established that people tend to rely heavily on projection for predicting others' behavior—that is, they use themselves as a model for prediction. This effect is especially strong when relatively little is known about the target [for a review, see Robbins and Krueger (2005)]. In general, then, it is likely that persuaders use the self as a model for argument generation: in other words, they argue in a way that they would themselves find convincing. This, in turn, suggests that the types of arguments used by persuaders can contain information relevant to understanding how they think about the issue at hand.

The tendency to use social projection is especially relevant in online settings. Much online discussion is either fully anonymous or conducted under pseudonyms, greatly limiting the amount of information available about the other party in a discussion. As such, we assume for the purposes of the present study that people will generally tend to use arguments that they themselves would find most convincing were they the audience rather than the persuader. This, in turn, should reflect the structure of their belief systems—the arguments that people find most convincing are those that match up with how they view the world (Darwin et al., 2011; Newheiser et al., 2011; Wood et al., 2012; Lewandowsky et al., 2013b). To that end, we systematically coded and analyzed conspiracist and conventionalist persuasive comments from four major news websites on articles relating to 9/11 from the period of July 1st through December 31st, 2011, encompassing the months surrounding the tenth anniversary of the attacks.

9/11 conspiracy theories provide an excellent research subject for several reasons. First, the community associated with these theories, known as the 9/11 Truth Movement, is noted for its substantial online presence and focus on Internet proselytizing. Bartlett and Miller (2011) observed that the movement's “mass membership backbone” (p. 45) devotes a substantial amount of time to producing large numbers of online comments, and Clarke (2007) saw the Truth Movement as a paradigmatic example of Internet conspiracy culture. Second, the timing was fortuitous, with the tenth anniversary of the 9/11 attacks, sure to herald a number of stories on the subject and therefore many relevant comments, having occurred shortly before data collection commenced. The recency of the materials lowered the probability of comments having been expunged from archives or lost as an unintended consequence of comment software upgrades. Third, just as the Truth Movement has a substantial online presence, so too do its conventionalist opponents in the skeptic movement. We therefore expected that there would be a good deal of debate between the two sides, providing further raw materials for analysis. Finally, the Truth Movement is a well-established community with a substantial intellectual output, including popular books (e.g., Griffin, 2004), conference circuits, several sub-organizations such as Architects and Engineers for 9/11 Truth, and at least one peer-reviewed journal, the *Journal of 9/11 Studies.* There is substantial debate within the Truth Movement regarding whether 9/11 was a controlled demolition, a deliberate intelligence failure, or even the result of exotic space-based weaponry (Barber, 2008). In short, its body of work is varied, voluminous, and well-developed, and should therefore be able to provide a wide range of different arguments for analysis.

If our reasoning regarding the influence of projection on persuasive tactics holds, we should see systematic differences in the characteristics of conspiracist and conventionalist arguments. Specifically, we should be able to replicate earlier results demonstrating that unrelated conspiracy beliefs are intercorrelated (e.g., Goertzel, 1994; Swami et al., 2010; Wood et al., 2012)—in this case, conspiracist comments should contain more positive (and fewer negative) references to unrelated conspiracy theories compared with conventionalist comments. Examining a long-standing correlate of conspiracy belief, we also investigated the degree to which comments contained explicit expressions of mistrust, predicting that conspiracist comments would be more likely to express mistrust of authorities or other targets than conventionalist comments (e.g., Wright and Arbuthnot, 1974; Simmons and Parsons, 2005). Further, we examined expressions of powerlessness, and predicted that conspiracist comments would express more concerns about power, as feelings of powerlessness have been shown to correlate reliably with conspiracy theory belief (Abalakina-Paap et al., 1999). Replicating the previously established relationships between conspiracy beliefs, trust, and power would increase confidence in the present study's methods and help to justify any novel results derived therefrom.

In addition to verifying the utility of this archival approach by replicating previous results, we made several novel predictions. First, if we are correct in our contention that much of the conspiracist worldview is based on a generalized rejection of official explanations rather than on positing particular alternative narratives, conspiracist comments should focus on refuting conventional explanations more than on presenting or supporting specific conspiracy theories. Therefore, conspiracist comments, relative to conventionalist comments, should be more likely to derogate rival explanations and less likely to promote their own. Second, we elected to examine the veracity of the long-held contention that “conspiracy theory” and “conspiracy theorist” carry an intellectual stigma (e.g., Bratich, 2002, 2008; Coady, 2006). If this is true, people should be unwilling to apply the term to themselves and should object when others do so. As such, we predicted that conspiracists would avoid applying the term “conspiracy theory” to their own beliefs (or “conspiracy theorists” to themselves), and would attempt to dispute others' usage of the term. While this might seem an obvious prediction—and indeed many authors take it as a given that the term is stigmatized—to our knowledge there have not yet been any empirical investigations of this contention.

Finally, another possible avenue by which the spread of conspiracy theories could be fruitfully understood is social influence theory (Latané, 1981). Since 9/11 conspiracy theories are (at least in the West) an opinion held by a vocal minority attempting to effect change, social influence theory (Latané, 1981) would predict that conventionalists, if they are good majority influencers, are more likely to show patterns consistent with normative social influence. In particular, Bratich (2008) has highlighted the hostility of intellectual orthodoxy toward conspiracist explanations for events and the labelling of conspiracists as paranoid or otherwise mentally ill (c.f. Hofstadter, 1964; Kalichman et al., 2010). At the same time, conspiracists are often hostile in a different way, dismissing conventionalists as naïve, gullible, and either unwitting dupes or willing stooges of the conspiracy (Crane, 2008; Byford, 2011). Therefore, we examined the hostility of each persuasive comment, whether characterized by outright insults, threats, dismissive sarcasm, accusations of complicity, or other hostile or insulting content.

## Materials and methods

### Articles

The raw data consisted of the comment sections of various online news articles. Samples were taken from news articles posted between July 1st and December 31st, 2011, on four mainstream news websites: ABC (American Broadcasting Company) News, CNN, the *Independent*, and the *Daily Mail*. This date range was chosen because of the large number of 9/11-related articles around the time of the tenth anniversary of the attacks, and these four news sites were selected on the reasoning that an ideal sample would not be restricted to a single country, journalistic style, or ideological position, as well as for more practical reasons such as search capabilities, comment archiving, and unpaid access.

Relevant articles were selected by searching for a series of terms within the specified date range: “9/11,” “11/9,” “September 11th,” “11th September,” “world trade center,” “world trade centre,” “wtc,” “al-qaeda,” “shanksville,” and “building 7.” Where possible (i.e., the *Mail* and *Independent*) the websites' own advanced search functions were used; on the remaining sites, we conducted the required searches using Google News.

### Comments

For each article that resulted from these searches, the public comment sections were read, and from these, we extracted verbatim all relevant comments regarding the 9/11 conspiracy theories. Specifically, since only persuasive comments were of interest, only comments containing original content that could be considered persuasive, or written with the intent to persuade, were extracted. Our analyses, for the most part, are predicated upon the idea that people will tend to project in order to construct persuasive arguments; non-persuasive comments, therefore, are written without regard to their perceived efficacy in convincing the other party (or neutral parties), as that is not their aim. To operationalize this constraint we adhered to four criteria.

1. The comment must not consist solely of insults, ridicule, or threats (e.g., “u stupid sheeple need 2 wake up lol,” “Let me know what your home address is, and we can have a frank “discussion” about your idiotic conspiracy theories”). This criterion was adopted because insults on their own are not persuasive, and while insults may be relevant to the hostility and stigma variables, they are irrelevant to the majority of the analyses we wished to conduct.
2. The comment must not consist solely of “meta” discussion (e.g., “I see the government disinfo machine is working overtime with all the shills posting here,” “can't believe CNN is letting these tinfoil hat nutjobs hijack a story about the 9/11 memorial”). As with insults, “meta” comments do not make persuasive arguments, and are in fact about entirely different subject matter—they are concerned with the minutia of discussion rather than with the conspiracy theories and conventional explanations in question.
3. The comment must not consist solely of a link to an external website, YouTube video, or similar, or a link with minimal description that adds no meaningful content (e.g., “go to ae911truth.org for some informed discussion about 9/11,” “google Popular Mechanics 9/11 debunking”). While it would be in principle possible to code the contents of such videos, websites, and other bodies of Web content, they are usually prohibitively large (particularly in the case of exhortations to conduct a Web search for a particular phrase, such as “Building 7,” that returns millions of results) and would require an entirely different set of coding criteria. Moreover, the linked content was uniformly the work of others rather than the commenter's own reasoning, as would be necessary for our reasoning concerning the link between argument generation, projection, and internal psychological representations to hold.
4. The comment must not be copied verbatim from an external source. This was determined by conducting web searches when a comment was extremely long, contained unusual formatting such as inappropriate line breaks, or was out of character in terms of word choice or grammatical ability for a previously recognized commenter. As with external links, these passages were not generated by the commenters and the projection line of reasoning therefore cannot be assumed to apply in this case as it would in the case of original arguments. As such, when an otherwise original post contained a passage quoted from an external source, only the original content was coded.

The author of each comment was recorded, along with the Web address of the parent news article and whether the comment was a direct reply to another, previously posted comment.

### Coding

Once the comments were collected, they were coded according to the hypotheses of interest. The tone of the comment (conspiracist or conventionalist) was of interest to all analyses, so this was the first content variable coded. Conspiracist comments were identified as any that either directly put forward a conspiratorial account of the events of 9/11, in whole or in part; that challenged the official account in a manner implying conspiracy or complicity among governments, intelligence services, corporations, occult associations, or secret societies; that otherwise favorably referenced common tropes of the 9/11 Truth Movement and its associated body of arguments, such as cryptic allusions to the fate of World Trade Center (WTC) Building 7, popular quotations from conspiracy websites or prominent theorists, and so on; that responded to conventionalist comments in a manner implying that the original commenter had the wrong impression in thinking that the attacks were perpetrated by agents of Al-Qaeda; or that was somewhat ambiguous in isolation but was written by a commenter previously observed to make conspiracist arguments or in the context of an argument or point made in the parent article that otherwise made the commenter's intent clear. Therefore, a comment on an article about a new book on 9/11 reading “Does the book explain how WTC7 imploded from fire, how a single passport was found intact within hours, how Bin Laden was in the American hospital in Dubai weeks before, how fighter jets were diverted 1000s of miles away, how NORAD was ordered to stand down… ” was coded as conspiracist. While this comment does not directly allege conspiracy, it refers obliquely to many common 9/11 conspiracist arguments and seems clearly intended to raise doubt regarding conventional explanations of 9/11.

Conversely, comments were coded as conventionalist if they explicitly endorsed or provided evidence in support of the mainstream account of 9/11 or another unofficial yet non-conspiracist explanation (such as Al-Qaeda independently planting bombs in the Twin Towers or bringing explosives onto the hijacked aircraft); if they argued against specific 9/11 conspiracy theories or conspiracist arguments such as those shown in the sample comment above; or, as in the case of conspiracist comments, if they were ambiguous in isolation but were written by a previously established conventionalist or in the context of a discussion thread or point made in the parent article that otherwise made clear the commenter's intent. For instance, the comment “LOL! Wow! What a conspiracy. Man, that tin foil hat has got to be tight today. Thousands of conspirators would be needed to pull off even a fraction of what you claim. And every one of them has been silent for almost 10 years now. Incredible…” was coded as conventionalist due to its argument that the conspiracy explanations of 9/11 are implausible and general mockery of conspiracists.

Since the first hypothesis concerned the number of unrelated conspiracy theories mentioned favorably and unfavorably in the comment, we coded two separate variables for each comment: one comprised the number of other conspiracy theories mentioned favorably, and the other comprised the number of other conspiracy theories mentioned unfavorably. Importantly, these counts did not include “superconspiracies,” or conspiracies that orchestrate other conspiracies (Barkun, 2006), of which 9/11 was thought to be a part. For instance, if a commenter accused the Bilderberg Group or the Illuminati of masterminding the 9/11 attacks, this would be considered part of the 9/11 conspiracy theory rather than a separate conspiracy theory entirely. However, if the commenter expressed the opinion that the Illuminati orchestrated both 9/11 and the JFK assassination, the latter would be included as an additional conspiracy theory.

The next hypotheses concerned trust and powerlessness. We therefore coded whether each comment contained expressions of mistrust, whether broadly or narrowly targeted (e.g., “never believe what the media tells you” or “nobody's trustworthy these days”), as well as powerlessness (e.g., “they've won, there's nothing we can do”).

Our primary hypothesis, and the one most relevant to the issue of conspiracist belief systems, concerned whether the comments contained positive or negative arguments. As such we coded for two separate binary-valued variables: first, whether the comment contained advocacy of the person's favored interpretation (e.g., “thermite residue in the wreckage is consistent with controlled demolition,” “office fires can burn hot enough, uncontrolled, to weaken structural steel to the point of collapse”); second, whether the comment contained derogation of the opposing interpretation (“there is no way that a plane would have left so little wreckage at the Pentagon,” “it's totally implausible that such a large conspiracy could be kept secret for so long”); and third, whether the comment directly put forward an explanation for either the entirety of 9/11 or an element of it (“9/11 was an inside job,” “the collapse was caused by terrorists flying planes into buildings, nothing more”).

We were also interested in how commenters used the term “conspiracy theory.” As such, we created a nominally-coded variable with values representing the different ways in which the comment used the phrase and its variations: not at all; applied to an opposing interpretation (“that's just a crazy conspiracy theory”); applied to the commenter's own interpretation (“it may be a conspiracy theory, but it's still true”); both; or disputed in its applicability (“calling something a conspiracy theory is just a way of silencing dissent”). This included variations on the term, such as “conspiracy theorist,” “silly conspiracy nonsense,” etc.

The final hypothesis concerned the degree to which persuasive conspiracist and conventionalist comments were hostile. As such, we coded the hostility of each comment toward those who hold opposing views on a scale of one (not at all hostile) to five (extremely hostile).

An example of how one post was coded on all variables is as follows:

“No it's you who needs to check his/her facts, Delft University burnt from the sixth floor to the top and only a portion of the building on north side collapsed, check out the photos of the building after the fire they're easy to find. They even managed to remove all of the books from the library on the first floor undamaged after the partial collapse. Nothing about the WTC buildings or Delft University's structures, fires, or collapse/partial collapse are comparable. As far as I can tell the WTC buildings are still the only large steel framed high-rise buildings to suffer total collapse due to fire, your poorly researched comment doesn't disprove the statement anyway.”

This comment, from a Daily Mail article, was part of a lengthy discussion regarding the plausibility of the WTC buildings collapsing due to fires and structural damage, as posited in the conventional explanation. This comment was made in response to a conventionalist who claimed that there is precedent for similar collapses and that the official explanation is therefore plausible. The comment claims that the WTC collapse and the example given by the conventionalist are not comparable, and emphasizes the unique (and therefore suspicious) nature of the WTC collapse. This comment was therefore coded as derogating rival explanations, but not advocating its own or directly posing a cause of the collapse. It contains no expressions of mistrust or powerlessness, mentions no other conspiracy theories, and does not mention the phrase “conspiracy theory” or any of its derivatives. However, it contains some hostility in the first and last sentences, giving it a hostility rating of two.

### Data preparation

While a wide variety of comments were obtained, certain serial commenters tended to dominate the conversation across several news articles and even multiple websites. The sample of 2174 comments contained 1156 unique authors, of whom 321 commented more than once. Therefore, in addition to analyzing the entire collection of comments, we conducted a separate analysis in which we calculated the mean values of each variable for each individual author and repeated the analysis on the level of authors rather than comments. All results obtained below were found at both the author and comment levels of analysis, which provides some assurance that any effects found are not the result of a few prolific commenters skewing the overall distribution of the data. For the sake of brevity, however, only the comment-level analysis is reported below.

### Inter-rater reliability

A random sample of 10% of the comments was coded by a second rater, and reliability analyses were conducted to determine the degree of concordance between the two raters. Cohen's kappa (κ) was used for all variables except hostility, the only ordinal variable, for which we instead calculated the intraclass correlation coefficient (ICC). Values of κ from 0.81 to 1.00 indicate almost perfect reliability, while reliability between 0.61 and 0.80 is considered “substantial” and from 0.41 to 0.60 “moderate” (Landis and Koch, 1977). Agreement regarding the conspiracist vs. conventionalist tone of each comment was high, κ = 0.84. As the reliability of the other variables used would depend heavily upon their classification as conspiracist or conventionalist, further reliability analyses were limited to those comments on whose tone both raters agreed. Accordingly, advocacy of the commenter's own explanation (κ = 0.64), derogation of the opposing explanation (κ = 0.61), and the usage of “conspiracy theory” (κ = 0.70) all showed substantial interrater reliability, while mistrust (κ = 0.49) and direct statements of what happened (κ = 0.55) showed moderate reliability (Landis and Koch, 1977). Hostility ratings were likewise acceptably reliable, ICC = 0.72. Finally, so few examples of powerlessness were found in the results that it was impossible to draw any conclusions regarding their relative prevalence in conspiracist and conventionalist comments. As such, reliability analyses were not performed on this variable.

## Results

Of the 2174 comments collected, 1459 were coded as conspiracist and 715 as conventionalist. The four news websites did not contribute equally to the sample, with 65 comments in 15 threads coming from ABC News, 632 in 29 threads from CNN, 1006 in 64 threads from the *Daily Mail*, and 471 in 27 threads from the *Independent*. Nevertheless, each site had approximately the same proportions of conspiracist and conventionalist comments—specifically, about twice as many conspiracist comments as conventionalist: for ABC, 21 conventionalist and 44 conspiracist; for CNN, 218 conventionalist and 414 conspiracist; for the *Daily Mail*, 330 conventionalist and 676 conspiracist; and for the *Independent*, 146 conventionalist and 325 conspiracist; χ^2^_(3)_ = 1.514, *p* = 0.68.

Table 1 shows the general results of the coding analysis. In line with our predictions, conspiracist comments mentioned more non-9/11 conspiracy theories as being correct than conventionalist comments [*M* = 0.12 per comment vs. *M* = 0.02; *t*_(2172)_ = 3.82, *p* < 0.001] and fewer such theories as being incorrect [*M* = 0.02 per comment vs. *M* = 0.18; *t*_(2172)_ = −7.51, *p* < 0.001]. Likewise, conspiracist comments were more likely to express mistrust than their conventionalist counterparts [10.6% vs. 1.4%; χ^2^_(1)_ = 57.22, *p* < 0.001]. We were unable to test the powerlessness prediction, however, as only two comments in the entire sample contained expressions of powerlessness.

**Table 1:** **Rhetorical components of conspiracist and conventionalist comments**.

	**Conspiracist**	**Conventionalist**
Mean conspiracy theories mentioned favorably	0.12	0.02
Mean conspiracy theories mentioned unfavorably	0.02	0.18
Mean hostility (1–5 scale)	1.43	2.07
% comments expressing mistrust	10.6	1.4
% comments advocating own explanation	31	56
% comments derogating other explanation	64	44
% comments explicitly providing a description of what happened	52	19
% comments describing own belief as a conspiracy theory	2.1	0.1
% comments describing opposing belief as a conspiracy theory	4.3	23.2
% comments describing both own and other beliefs as conspiracy theories	0.3	0
% comments disputing usage of “conspiracy theory”	4.5	0.1

Analysis revealed a number of differences between the rhetorical styles of conspiracist and conventionalist commenters. Thirty-one percent of conspiracist comments contained information that constituted support for their own position, compared to 56% of conventionalist comments. This difference was significant, χ^2^_(1)_ = 121.69, *p* < 0.001. In contrast, 64% of conspiracist comments involved derogation of the opposing explanation, significantly more than the 44% of conventionalist comments that did the same, χ^2^_(1)_ = 80.13, *p* < 0.001. Unexpectedly, while only 19% of conventionalist comments directly put forward an explanation for the events of 9/11, 52% of conspiracist comments did so, χ^2^_(1)_ = 53.56, *p* < 0.001.

Conventionalist comments (*M* = 2.08, *SD* = 1.02) were significantly more hostile than conspiracist comments (*M* = 1.44, *SD* = 0.79), *t*_(2172)_ = 16.22, *p* < 0.001 (see Table 1). Finally, neither conspiracists nor conventionalists were particularly willing to self-apply the term “conspiracy theory” or its derivatives: only 31 conspiracist comments referred to their beliefs as such, while 63 used the term to describe the official story of 9/11, four used it to describe both theories, and 65 disputed others' use of it. Conventionalists were likely to call opposing beliefs conspiracy theories, with 166 doing so, compared to only a single comment that self-applied the term and another one that contested its applicability. No conventionalist comments called both explanations “conspiracy theories.”

## Discussion

The data were generally consistent with our predictions. Conspiracist comments expressed more favorable opinions about unrelated conspiracy theories than conventionalist comments did. This serves as a conceptual replication of previous findings indicating that beliefs in conspiracy theories tend to be correlated: if someone agrees with 9/11 conspiracy theories, they are also more likely to agree with other conspiracy theories (e.g., Goertzel, 1994; Swami et al., 2010, 2011; Wood et al., 2012). Further, in accordance with previous work on the role of trust in conspiracy theory beliefs (e.g., Wright and Arbuthnot, 1974; Abalakina-Paap et al., 1999; Simmons and Parsons, 2005), conspiracist comments were more likely to contain expressions of mistrust than were conventionalist comments. Despite the unexpected impossibility of testing the powerlessness hypothesis, this cluster of results should increase confidence in the validity of the remainder of the present study's conclusions. The well-established tendencies for conspiracists to be less trusting than average and for conspiracy theory beliefs to intercorrelate have manifested themselves in the persuasive communications examined, which suggests that other tendencies may do so as well.

Most notably, and in accordance with the idea that opposition to officialdom is a major component of the conspiracist belief system, conspiracy advocates showed a tendency to spend much more time arguing against the official explanation of 9/11 than advocating an alternative. Conspiracy opponents showed the opposite pattern, advocating their own explanation more than they argued against the opposing one. This pattern of results supports the idea that conspiracy theories have their basis more in opposition to officialdom than in beliefs in specific alternative theories (Dean, 2002; Wood et al., 2012). For the adherents of the 9/11 Truth Movement examined here, the search for truth consists mostly of finding ways in which the official story cannot be true. There is much less of a focus on defending coherent explanations that can better account for the available evidence. However, conspiracists were more likely to provide direct explanations for the events of 9/11 than their conventionalist counterparts were—for instance, it was more common to see a comment saying “9/11 was an inside job” or “WTC7 was demolished” than “9/11 was done by terrorists” or “WTC7 collapsed because of fires and structural damage.” This seems like a paradoxical pattern, but conspiracist comments often simply stated that 9/11 was an inside job as a sort of slogan without much to support it. Many other comments took the form “the official story is impossible, therefore 9/11 was the result of a conspiracy.” For instance, one representative comment from a CNN article read, “Inside Job 9/11! If it was a real terrorist attack U.S. military would have blew up the planes while in the air before they could hit any population area!” Furthermore, many of the news articles on which the comments appeared featured the official explanation of 9/11 in some detail, meaning that it may have been less necessary for conventionalists to summarize the conventional account themselves.

We also found that hostility was higher in persuasive arguments made by conventionalists than in those by conspiracists. As 9/11 conspiracism is by and large a minority viewpoint in the West (WorldPublicOpinion.org, 2008), this makes sense: conventionalists, rather than focusing on presenting novel information, instead attempt to enforce conformity to the majority viewpoint (Latané, 1981). While the inter-rater reliability for hostility was good, there is a risk that we may not have captured the full spectrum of responses, as we specifically excluded comments that consisted solely of threats, insults, or ridicule. As such, although we cannot say with certainty that conventionalist comments are more hostile on average than conspiracist comments, we can say with some confidence that this is true among comments that also contained some amount of persuasive content.

Finally, the statistics on the usage of the phrase “conspiracy theory” provide an instructive illustration of how the term is viewed. Few people were eager to apply it to their own positions. Conspiracists were more likely to apply it to the conventional narrative, often counterintuitively referring to it as “the official conspiracy theory,” or to dismiss the term as needlessly loaded and derogatory, consistent with recent scholarly characterizations (Bratich, 2008). Part of the problem is likely to be the vagueness of the term; while we have provided a working definition in the present study, there is no universal agreement on what exactly constitutes a conspiracy theory (Coady, 2006). Clearly, however, the prevalence of counter-argumentation to the use of the label by others points to some disdain for the term among conspiracists.

There are other possible interpretations for some of these results. For instance, the observed difference in the usage of other conspiracy theories between conspiracist and conventionalist comments could be seen as an issue of rhetorical congruence more than of genuine belief. This pattern could naturally arise as the result of an inclination toward arguing by analogy: conspiracists might compare the 9/11 attacks to the JFK assassination, which a majority of Americans believe was the result of a conspiracy (Goertzel, 1994), in order to make a conspiracy theory seem more plausible. In contrast, conventionalists could compare 9/11 conspiracy theories to more overtly implausible examples, such as the proposed cover-up of the existence of Bigfoot or the idea that Elvis Presley is still alive, in order to make the point that conspiracy theories in general are not to be taken seriously. Indeed, a *post-hoc* examination of the data revealed that 23 comments mentioned the JFK assassination conspiracy theory favorably, while only nine mentioned it negatively. The 7/7 bombing conspiracy theories showed a similar pattern, with 6 negative mentions to 16 positive. Other theories, such as those concerning the moon landing (27 negative, 6 positive), Elvis (21 negative, 0 positive), aliens (20 negative, 2 positive), and David Icke's reptilian shapeshifters (8 negative, 1 positive), showed the opposite pattern. Not all of these mentions followed the general pattern evident in the data; some conventionalists said that while some other conspiracy theories are true, there is no evidence for a 9/11 conspiracy, and some conspiracists claimed that while most conspiracy theories are bogus, in the case of 9/11 the evidence is sufficient to reject the official story. This form of argument might ultimately be persuasive: people who portray themselves as nominal conventionalists who nevertheless, find 9/11 conspiracy theories plausible are essentially portraying themselves as deviant ingroup members. Such people can be very effective in exerting social influence on the majority (e.g., Maass and Clark, 1984).

Ideas of rhetorical congruency and self-presentation recall the issue of whether people's persuasive communications are really an accurate reflection of their own thoughts and ideas rather than a carefully calculated attempt to engage with others' biases and reasoning. The 9/11 Truth Movement is, by and large, a movement of converts—most “Truthers,” at some point, became convinced that their previous belief in the official story was wrong (Kay, 2011). Therefore, in debating with those who hold the positions they previously held, they might repeat the arguments that first caused them to doubt the conventional narrative and shaped their subsequent thinking accordingly. On the other hand, the actual content that the discussions centered upon was often highly technical, and many of the arguments were unlikely to have been generated entirely by the people doing the commenting. While some commenters made intuitive judgments about the physics of crashing airplanes and collapsing buildings, many others relied on arguments advanced in websites or documentaries devoted to either advancing or debunking 9/11 conspiracy theories. With the amount of information to choose from, however, the arguments commenters chose to put forward may still reveal useful information about their own decision-making.

While the results of the present study fit with previous work on belief and disbelief in conspiracy theories, some of the novel results found here would benefit from confirmation via other methodological approaches. If conspiracist beliefs are generally structured in the way we posit, it should be observable under experimental conditions—for instance, people with a conspiracist worldview might find a piece of evidence to be more convincing if it is presented as a refutation of the official account of some event rather than as proof of a specific conspiracy theory. Likewise, while we have confirmed that “conspiracy theorist” is not a well-liked term among conspiracists, we have not investigated its impact—if the term is used to describe a certain account of an event, the negative associations of it might reduce the perceived plausibility of the argument.

In sum, our results are in agreement with predictions derived from prior research. Consistent with much of the existing literature on individual differences associated with conspiracy belief, comments that supported 9/11 conspiracy theories were more likely to express mistrust and to refer to other conspiracy theories favorably. Conspiracists were less overtly hostile than their conventionalist counterparts, and did not appreciate being called conspiracy theorists. Perhaps most importantly, however, the finding that conspiracists spend more time arguing against official explanations than for alternative explanations supports the idea that the conspiracy worldview is based more on disbelief than on positive belief. The coherence of the conspiracist belief system is driven by higher-order considerations such as a disbelief in official narratives, rather than positive beliefs in particular alternative narratives. This result also agrees with previous informal observations by conventionalist commentators, who devote a great deal of time to examining and debunking conspiracy theories. One tactic which conventionalists often accuse conspiracists of using is “anomaly hunting”:

They imagine that if they can find (broadly defined) anomalies in that data that would point to another phenomenon at work. They then commit a pair of logical fallacies. First, they confuse unexplained with unexplainable. This leads them to prematurely declare something a true anomaly, without first exhaustively trying to explain it with conventional means. Second they use the argument from ignorance, saying that because we cannot explain an anomaly that means their specific pet theory must be true. I don't know what that fuzzy object in the sky is—therefore it is an alien spacecraft (Novella, 2009).

The observed tendency of conspiracy theory advocates to argue against conventional narratives rather than in favor of particular alternatives closely resembles this description of anomaly hunting, and also parallels Keeley's (1999) observation that conspiracy theories rely heavily on “errant data” rather than on crafting coherent alternative explanations (p. 117). We argue that in fact, anomaly hunting, or a fixation on errant data, is a manifestation of the way conspiracism is structured as a worldview. In general, conspiracy belief is not based around specific theories of how events transpire, though these may exist as well. Instead, conspiracism is rooted in several higher-order beliefs such as an abiding mistrust of authority, the conviction that nothing is quite as it seems, and the belief that most of what we are told is a lie. Apparent anomalies in official accounts seem to support this, even if they do not point to a specific, well-defined alternative. For many conspiracists, there are two worlds: one real and (mostly) unseen, the other a sinister illusion meant to cover up the truth; and evidence against the latter is evidence for the former.

### Conflict of interest statement

The authors declare that the research was conducted in the absence of any commercial or financial relationships that could be construed as a potential conflict of interest.
